# Microstructural Modification and Characterization of Sericite

**DOI:** 10.3390/ma10101182

**Published:** 2017-10-16

**Authors:** Yu Liang, Hao Ding, Sijia Sun, Ying Chen

**Affiliations:** School of Materials Science and Technology, China University of Geosciences, No. 29 Xueyuan Road, Haidian District, Beijing 100083, China; liangyuaadd@gmail.com (Y.L.); 1012122105@cugb.edu.cn (S.S.); chenying@cugb.edu.cn (Y.C.)

**Keywords:** sericite, thermal modification, acid activation, sodium modification

## Abstract

Activated sericite was prepared by thermal modification, acid activation and sodium modification, and it was characterized by X-ray diffraction (XRD) analysis, differential scanning calorimetry (DSC), N_2_ adsorption test, thermo-gravimetric analysis (TGA), nuclear magnetic resonance (NMR), and scanning electron microscope (SEM). The results indicated that the crystallinity of raw sericite decreased after thermal modification; the pores with sizes between 5 nm to 10 nm of thermal-modified sericite have collapsed and the surface area increased after thermal modification. The dissolving-out amount of Al^3+^ reached ca. 31 mg/g in the optimal processing conditions during acid activation; cation exchange capacity (CEC) of acid-treated sericite increased to 56.37 mmol/100 g meq/g after sodium modification compared with that of raw sericite (7.42 mmol/100 g). The activated sericite is a promising matrix for clay-polymer nanocomposites.

## 1. Introduction

In recent years, great attention has been paid to clay-polymer nanocomposites due to their extraordinary properties. Compared with pure polymer, this category of composites usually exhibits higher moduli [1,2,3], larger strength and heat resistance [4], smaller gas permeability [5,6,7], better fire retardancy [8,9], higher ionic conductivity [10], and increased biodegradability of biodegradable polymers [11,12,13]. Clay-polymer nanocomposites are widely used in a range of key areas, such as aerospace, automobile, appliances, and electronics [14,15,16]. These properties depend heavily on the structure of nanocomposites, which is determined by the physical properties of clay mineral, such as its cation exchange capacity (CEC) which is used to quantify the excess negative charge of layered silicates and their capability to exchange ions. CEC is highly dependent on the nature of the isomorphous substitutions in the tetrahedral and octahedral layers. 

The common layered silicates used for preparation of clay-polymer nanocomposites are 2:1 type (montmorillonite, vermiculite, and mica) and 1:1 type (kaolinite); the former is used much more frequently. Montmorillonite [17,18,19,20,21,22] and vermiculite [23,24,25,26,27,28,29] have been mostly investigated as the matrix materials for clay-polymer nanocomposites because of their swelling behavior and ion exchange properties.

Although sericite belongs to 2:1 clay minerals, it does not swell in water and has almost no ion exchange capacity. It can hardly be intercalated because it has a high layer charge density close to 1.0 equivalent per O_10_(OH)_2_, which produces pretty strong electrostatic force [30]. This layer charge stems mainly from the substitution of Al^3+^ for Si^4+^ in tetrahedral sheet. Sericite is very fined squamous-structured muscovite, as one kind of mica family. It holds the advantages of high moduli, stable chemical property, high electrical insulation and good ultraviolet ray resistance [31,32,33,34]. Therefore, it is necessary to explore the preparation of an expandable sericite with relatively high CEC.

The purpose of activation is to permanently reduce the layer charge of sericite and obtain a number of exchangeable cations. The physical and chemical modifications have long been used to activate clay and clay minerals, such as acid activation and thermal treatment. Salt modification, mechanical grinding [35,36] and swelling by the decomposition of hydrogen peroxide [37] are also usually employed. Poncelet and del Rey-Perez-Caballero [25,38] permanently reduced the global negative charge of the mineral layers by the combination of calcination and acid activation, and the resulting product (activated vermiculite/phlogopite mica) was successfully used as matrix in micro porous 18 Å Al-pillared nanocomposites. They found that Al^3+^ in tetrahedral sheet could be partly dissolved out by the combination process and yet had an unremarkable effect on the structure of octahedral sheet. Furthermore, a well-swelled sericite with 80% exchangeable K^+^ and a CEC of 110 mmol/100 g was obtained by Shih and Shen [32] by thermal modification and Li-hydrothermal treatment.

In this study, activated sericite was prepared by thermal modification, acid activation, and sodium modification. The X-ray diffraction (XRD) analysis, N_2_ adsorption test, nuclear magnetic resonance (NMR) and scanning electron microscope (SEM) were used to elucidate the effect of this process. From our study, it can be seen that thermal modification can reduce the layer charge and crystallinity of sericite. Acid activation can dissolve both the octahedral and tetrahedral Al^3+^ out and reduce its layer charge and sodium modification can finally improve the CEC value of sericite. Therefore, the whole modification process can make sericite more suitable for polymer-clay nanocomposites.

## 2. Materials and Methods

### 2.1. Materials

The raw sericite (S_0_) was obtained from Anhui province, China. Its mean size is about 10 μm. CEC of S_0_ is 7.42 mmol/100 g (0.07 meq/g). The quantitative analysis of the raw material showed that the purity of raw sericite is 93.2%, with 6.8% of quartz. Ruling out the influence of quartz, the chemical composition of S_0_ is listed in Table 1. The chemical formula is (K_0.79_Na_0.11_Ca_0.01_)(Al_1.64_Ti_0.02_Fe_0.18_Mg_0.24_)(Al_0.92_Si_3.08_)O_10_(OH)_2_.

### 2.2. Preparation

A certain amount of raw sericite (S_0_) was put into Al_2_O_3_ crucibles and heated between 500 and 1000 °C in muffle for 1 to 3 h and cooled to room temperature naturally. After that, the thermally-treated product (S_1_) was stirred with different kinds and concentrations of acid between 60 to 95 °C in thermostatic water bath for 4 h. The acid-treated product (S_2_) was washed, filtrated, and dried at 80 °C. In this study, experiment term was based on an orthogonal term array experimental design (OA (9, 3^4^)) where the following four variables were analyzed: the kinds of acid (factor A), acid concentration (factor B), reaction temperature (factor C) and reaction time (factor D). Finally, sodium chloride was added to react with S_2_ in round bottomed flask. The sodium modified product (S_3_) was obtained by mixing, washing, centrifuging, and drying at 80 °C. The orthogonal experiment term method (OA (9, 3^3^)) was used to find three optimal parameters: concentration of Na^+^ (factor A), reaction temperature (factor B) and reaction time (factor C).

### 2.3. Characterization 

The X-ray diffraction patterns were obtained on a Rigaku Rotaflex X-ray powder diffractometer (Rigaku, Tokyo, Japan), employing Cu Kα radiation, 40 kV, 100 mA. The X-ray diffraction (XRD) patterns in the 2θ range from 3°–70° were collected at 4°/min. Simultaneous collection of DSC and TGA signals was carried out using a SDT Q600 analyzer (TA, New Castle, DE, USA) under air flow and heated from room temperature to 1100 °C at 10 °C/min. The BET surface area of the samples was determined by N_2_ adsorption by using NOVA4000 equipment (Quantachrome, Boynton Beach, FL, USA). Prior to N_2_ adsorption, the samples were evacuated at 473 K under vacuum for 4 h. The pore size distribution was calculated using the BJH method. ^27^Al NMR spectrum (130.327 Hz) was recorded on a Bruker Avance III spectrometer (Bruker, Karlsruher, Germany). The dwell time is 0.01 s and the rotational speed is 6000 rpm. 

## 3. Results and Discussion

### 3.1. Thermal Modification

**Figure 1:**
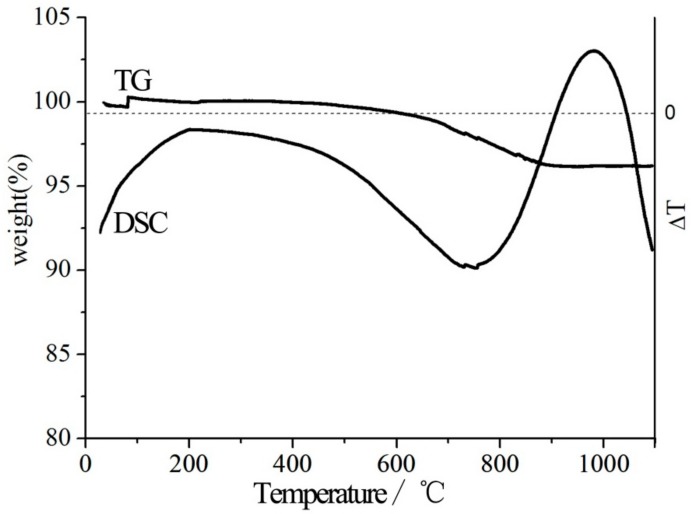
TG and DSC curves of S_0_.

A slight weight loss is observed in the TG curve at low temperature (Figure 1), which is attributed to absorbed surface water. There is a mass loss in the TG curve at 3% between 670 and 841 °C according to the DSC curve peak at the same temperature, which indicates that the hydroxyl groups were lost with increasing temperature during thermal modification.

**Figure 2:**
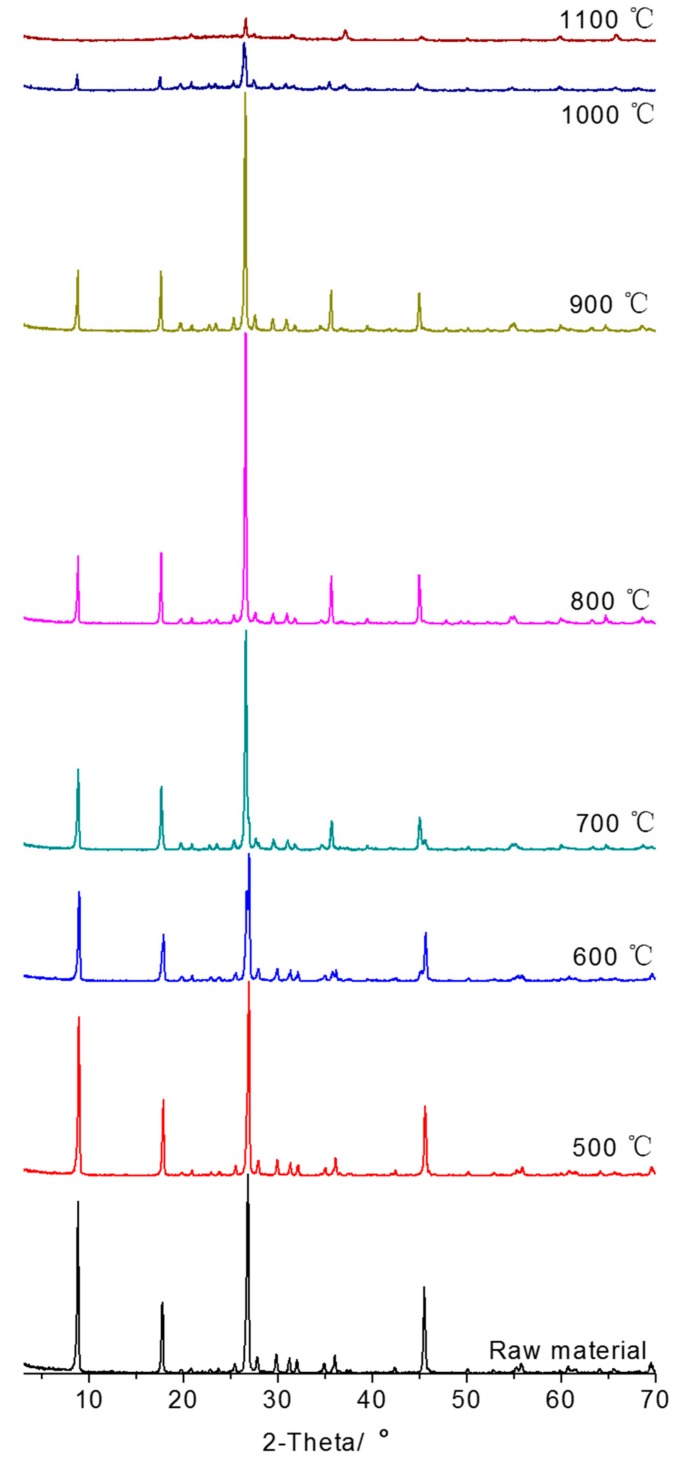
XRD patterns of sericite after heating at different temperatures.

The XRD patterns of sericite which were activated at different temperatures are shown in Figure 2. The intensities of major reflections decreased gradually as the temperature increased and finally almost disappeared at 1100 °C, which suggested that the mica-type phase persisted when the temperature was lower than 900 °C. The phase transformation would occur and its crystal integrity would be destroyed gradually when heated at 1000 °C. The lattice activated degree can be judged by lattice distortion level. The relationship between lattice distortion level, crystal size, full width at half maximum (FWHM) of reflections and diffraction angle can be calculated by Scherrer’s equation:

Bcos θ = Kλ/D + 4Δdsin θ/d(1)

where B is FWHM; θ is diffraction angle; K is the form factor, which is close to 1; λ is the wavelength of X-ray; D is the crystal lattice size; d is the distance between crystal planes; Δd is the average deviation between the distance of reflecting planes under study and the mean value d. 4Δd/d shows the level of lattice distortion. The larger the value, the higher the distortion level, and vice versa. In this part, Bcos θ and sin θ of different products which reflect the lattice distortion level were calculated from XRD data. The slope (4Δd/d) and intercept (kλ/d) were obtained by linear fitting, using sin θ as *X* axis and Bcos θ as *Y* axis. The obtained values were summarized in Table 2.

As shown in Table 2, the value of 4Δd/d of raw sericite is 0.0118 (close to 0), which is a proof of little lattice distortion. As the temperature increased, the absolute value of 4Δd/d of the thermally-treated product increased gradually, reaching the largest value at 800 °C. The line’s slope starts to decrease at 900 °C, follows by an even larger decrease at 1000 °C. The results above demonstrate that the best activated temperature is 800 °C (with the same holding time). The loss of crystallinity is evaluated using the FWHM index (Table 3). Heating at 800 °C got the largest FWHM, which is a sign of the most lattice defects and distortion. 

**Figure 3:**
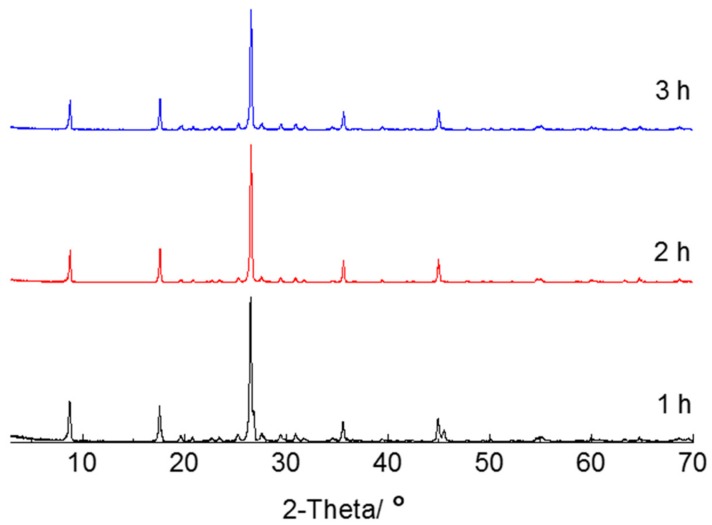
XRD patterns of sericite with different holding time at 800 °C.

The raw sericite samples were heated at 800 °C and preserved for 1 h, 2 h, and 3 h. The XRD patterns are shown in Figure 3. The values of kλ/D and 4Δd/d extracted from the XRD data are shown in Table 4. The absolute value of 4Δd/d of sericite with a holding time of 1 h is much higher than that of raw material. When the holding time increased to 2 h or more, the slope decreased. The potential reason is that the increasing holding time make the unobvious preferential orientation of flakes caused by lattice distortion increase. Therefore, the best preservation time is 1 h.

**Figure 4:**
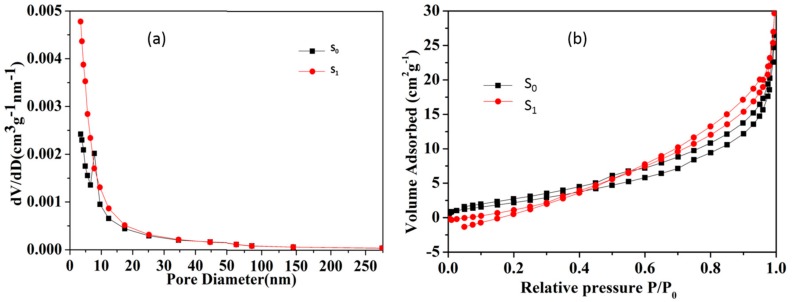
(**a**) Pore diameter distribution of raw sericite (S_0_) and thermal-activated sericite (S_1_); (**b**) the isotherm of N_2_ adsorption-desorption on sericite before and after thermal modification.

N_2_ physisorption measurements have also been performed on both S_0_ and S_1_ (Figure 4). It can be seen that after thermal modification, the pores with sizes between 5 nm to 10 nm of thermally-modified sericite have collapsed. The surface area of S_0_ is 14.653 m^2^/g, while the surface area of S_1_ is 16.579 m^2^/g, which means thermal modification increased the activity of sericite.

### 3.2. Acid Activation

According to the research of Poncelet and del Rey-Perez-Caballero [25] on the activation of vermiculite and phlogopite, the combination of acid treatment and heat treatment was employed to modify the microstructure of sericite. As a result, Al^3+^ was dissolved out and the negative layer charge was reduced, which enables sericite to take on an ion exchange capacity. The results of acid activation were evaluated by dissolving-out an amount of Al^3+^. The larger the dissolving-out amount of Al^3+^, the better the effect of acid activation.

The main four factors, kinds of acid (factor A), acid concentration (factor B), reaction temperature (factor C), and reaction time (factor D) were researched and each control parameter has three experimental levels (Table 5) [39,40].

The optimal values of different factors determined with reference to Table 5 are as follows: nitric acid, 5 mol/L, 95 °C, 5 h. In addition, the factors’ levels of significance are as follows: reaction temperature > acid concentration > reaction time > type of acid.

**Figure 5:**
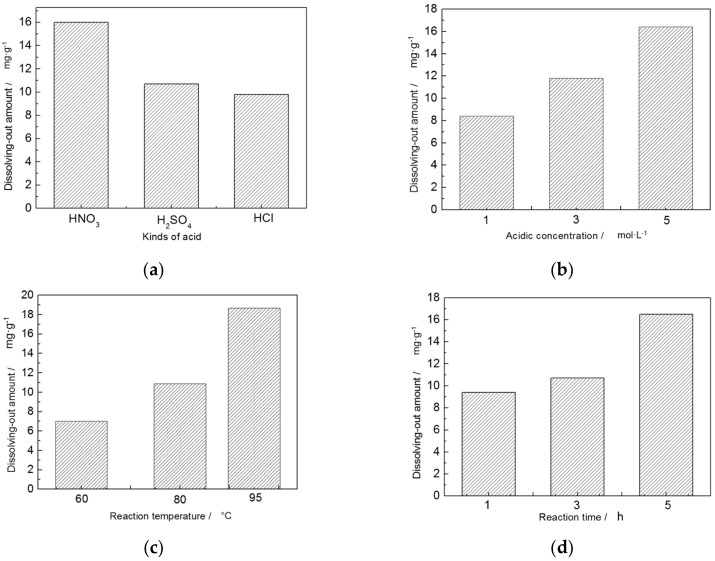
Effect of (**a**) kinds of acid; (**b**) acid concentration; (**c**) reaction temperature; and (**d**) reaction time on dissolving-out amount of Al^3+^.

Figure 5 shows the single effect of each factor on acid activation. A higher reaction temperature helped to dissolve Al^3+^ out. When temperature was low, the reaction system could not obtain enough power, so only a small amount of Al^3+^ dissolved out. The dissolving-out amount of Al^3+^ increased with the acid concentration, which means the higher the H^+^ concentration, the better the result. However, when the acid concentration is ultrahigh, the layered structure would, conceivably, be seriously destroyed, which is not good for activation of sericite. Additionally, nitric acid is more effective on acid activation than the other two, although the kind of acid is not the most significant factor. 

**Figure 6:**
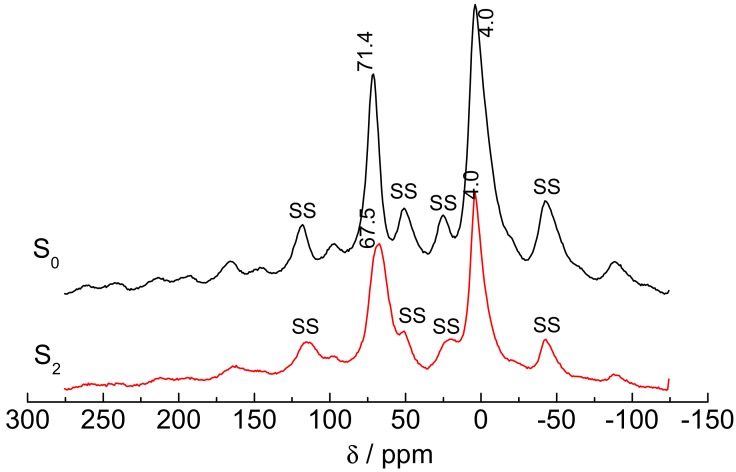
^27^Al NMR spectrums of S_0_ and S_2_ (SS means “spinning sidebands”).

NMR analysis was done after acid activation. The range of chemical shift (δ) of Al is 450 ppm. Generally, δ of octahedral Al (Al_o_) species and tetrahedral Al (Al_t_) species is −10 to 10 ppm and 50–70 ppm, respectively. Therefore, ^27^A1 NMR is employed to distinguish the two kinds of Al in clay. As shown in Figure 6, δ of Al_t_ and Al_o_ of S_0_ was 71.4 (spinning sidebands were 118 and 25, respectively), and 4.0 (sidebands were 50 and −42, respectively), both of which were similar to theoretical values. The counterparts of S_2_ turned to be 67.5 and 4.0, respectively.

The sharp peaks in ^27^A1 NMR is usually the sign of short range order in Al, while broad peaks signal short range disorder. The peaks of S_0_ in ^27^Al NMR spectrum are sharper than those of S_2_, which indicate that the layered structure become more disordered. The peak intensities of Al_t_ and Al_o_ of S_2_ decrease by 34% and 32%, respectively, as compared with those of S_0_, which suggest that Al_t_ and Al_o_ are both dissolved out.

The relative content of Al_t_ in S_2_ increased, and yet the peak width increased, which signaled an uneven distribution of Al_t_. On the contrary, the relative content of Al_o_ decreased, and yet the peak width decreased, which is a sign of even distribution of Al_o_. This phenomenon can be explained by the decrease of layer charges that leads to a higher order degree of Al_o_ [41]. The ratio of Al_t_ to Al_o_ decreased after acid treatment from 6.25:10 to 5.82:10, which means acid-treated sericite is more suitable for the ion exchanges in the next step.

### 3.3. Sodium Modification

**Figure 7:**
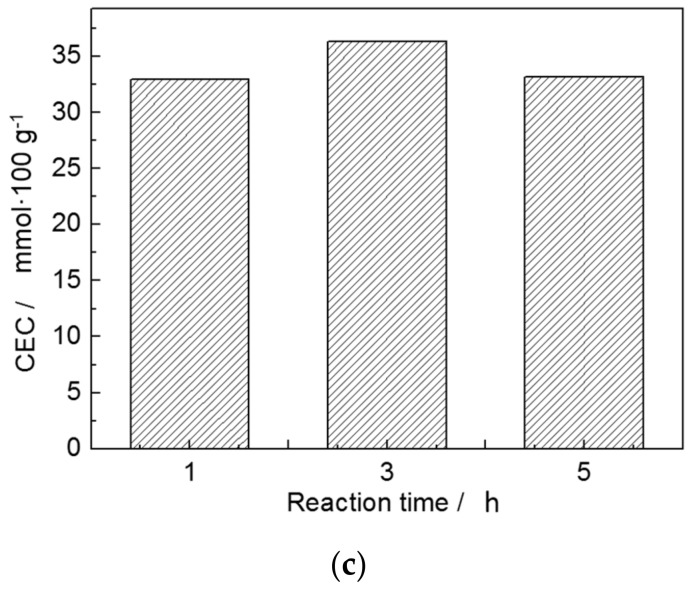

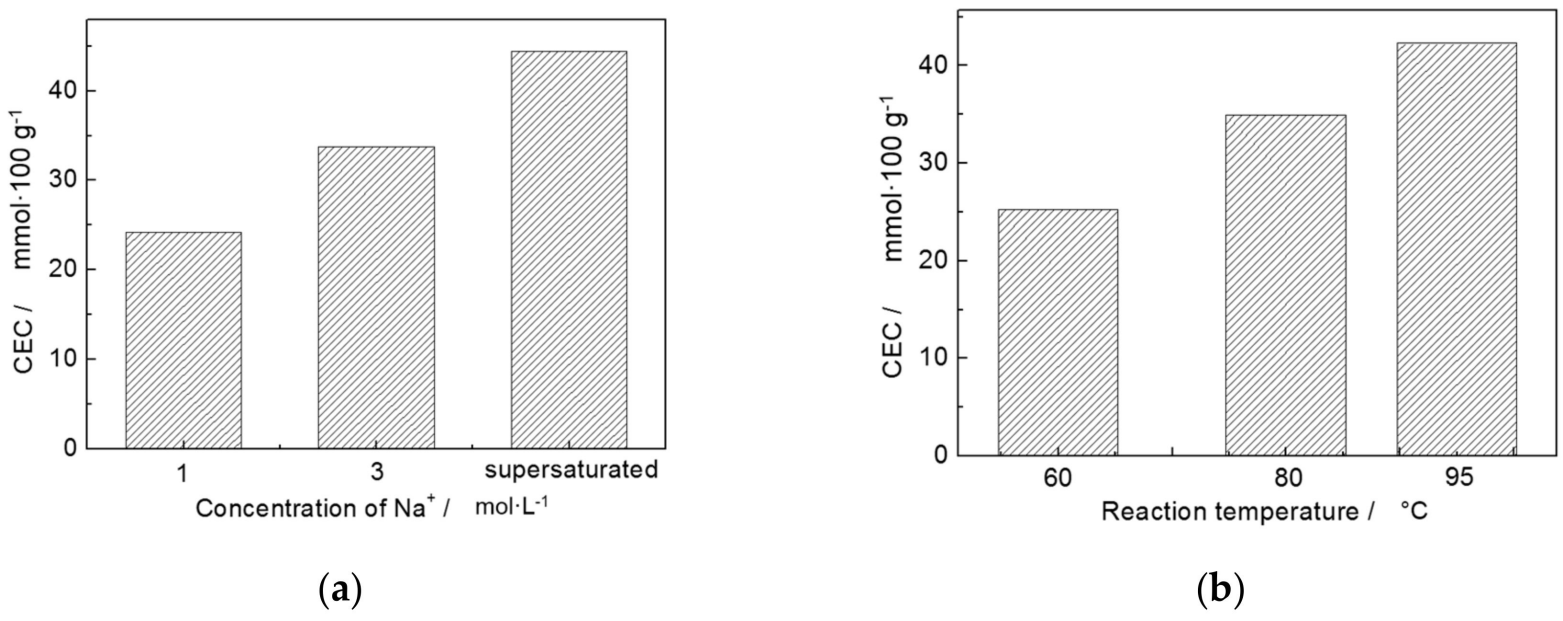
Effect of (**a**) concentration of Na^+^; (**b**) reaction temperature; and (**c**) reaction time on CEC.

The results of sodium modification are evaluated by CEC. The larger the CEC value, the better the sodium modification result. Detailed sodium modification conditions are listed in Table 6. It can be seen that the optimal sodium modification conditions are as follows: supersaturated solution of sodium chloride, 95 °C, 3 h. The factors’ levels of significance are as follows: Na^+^ concentration > reaction temperature > reaction time. The single effect of each factor on sodium modification is shown in Figure 7, which indicates that higher concentration of Na^+^ and higher reaction temperature are of great benefit to the CEC of S_3_.

The interlayer potassium cation twelve coordinates with two aspectant hexagonal holes created by the Si/Al tetrahedral sheet, and is able to fit the two holes very tightly between the layers. Therefore, potassium cation and two adjacent tetrahedron sheets are bonded together closely by the electrostatic attraction. Consequently, it is hard for K^+^ to be exchanged by Na^+^. However, the combination of thermal modification and acid activation made the exchange possible, which was due to the activation of lattice. Na^+^ has the superiority of the smaller hydrated ionic radius and lower hydrated energy compared with those of K^+^. The higher reaction osmotic pressure was applied by the higher concentration of Na^+^, and higher reaction temperature made ions turn to be more active. Therefore, higher concentration of Na^+^ and reaction temperature would benefit sodium modification. Additionally, the exchange of Na^+^ for K^+^ was in the state of dynamic equilibrium. Therefore, longer reaction time has no effect on the CEC of S_3_. Compared our study with the study of Shih, we used different treatment methods (we used Na-hydrothermal treatment while they used Li-hydrothermal treatment) at different temperatures (we used 60–95 °C while they used 90–270 °C). This is why our final CEC value (56.37 mmol/100 g) is lower than theirs (110 mmol/100 g). 

**Figure 8:**
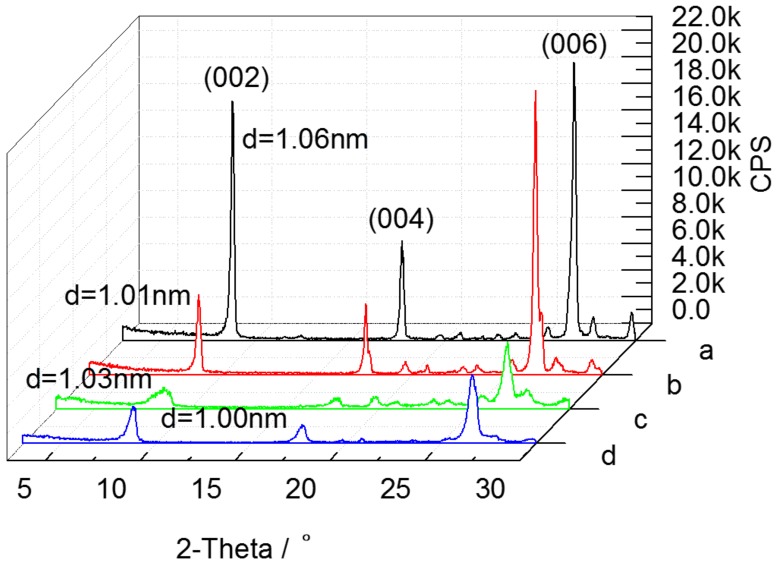
XRD patterns of (**a**) S_0_; (**b**) S_1_; (**c**) S_2_; and (**d**) S_3_.

The XRD patterns of raw material and activated products (S_1_, S_2_, and S_3_) prepared at the optimal conditions are shown in Figure 8, and the loss of crystallinity is evaluated using the FWHM index (Table 7). The decrease of reflection intensities of S_1_ was caused by the removal of the hydroxyl water of raw material corresponding to the increase of FWHM of S_1_. After acid activation, the reflection intensities and crystallinity of S_2_ further decreased, which was a sign of more lattice defects and larger lattice distortion. Compared with the pattern of S_2_, the interlayer space of S_3_ decreased slightly, which was due to the exchange of Na^+^ for K^+^ between layers. However, sodium modification led to a better crystallinity of S_3_ than that of S_2_. This may be due to the fact that Na^+^ balanced the change of layer charge of S_2_ caused by acid activation, and the crystal structure of S_2_ was repaired to some extent.

**Figure 9:**
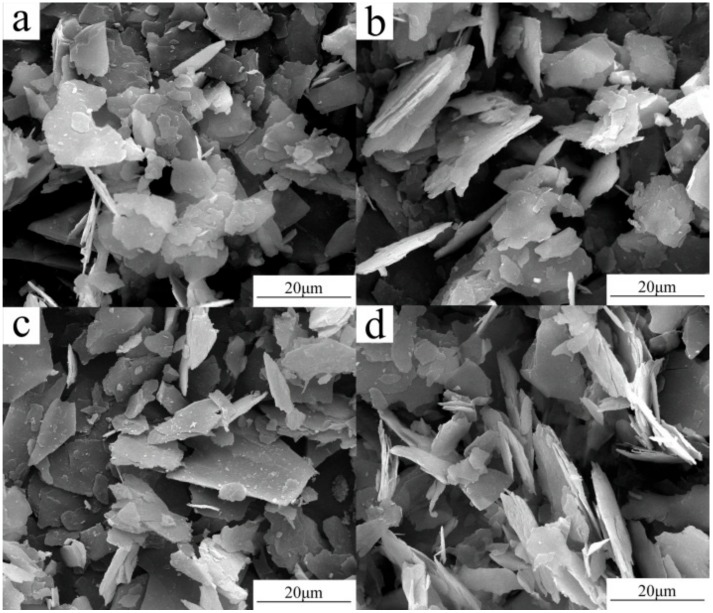
SEM images of (**a**) S_0_; (**b**) S_1_; (**c**) S_2_; and (**d**) S_3_.

From SEM images, it can be seen that S_0_ has smooth surfaces, sharp fringed flakes, and uniform particle size (Figure 9). The SEM images of S_1_, S_2_, and S_3_ clearly indicate that the mica-type phase of sericite persists while the particle surfaces become rougher. 

## 4. Conclusions

Activated sericite was prepared by thermal modification, acid activation, and sodium modification. The final product can be prepared by heating at 800 °C for 1 h, reacting with 5 mol/L nitric acid at 95 °C for 5 h and mixing with supersaturated solution of sodium chloride at 95 °C for 3 h. After modification, the mica-type phase persisted while its crystallinity decreased. The CEC of the final product can be enlarged from 7.42 mmol/100 g to 56.37 mmol/100 g meq/g. The activated sericite is much more suitable than raw sericite to prepare polymer-clay nanocomposites.

## Figures and Tables

**Table 1:** Chemical composition of the original sericite.

Composition	SiO_2_	Al_2_O_3_	Fe_2_O_3_	TiO_2_	K_2_O	Na_2_O	CaO	MgO	SO_3_	L.O.I	Total
Content (mass %)	45.71	28.32	3.04	0.35	8.09	0.71	0.10	2.12	0.075	4.47	99.555

**Table 2:** Relation between Bcosθ and sinθ of sericite activated at different temperatures in the (002) reflection.

Temperature (°C)	Raw Material	500	600	700	800	900	1000
kλ/D	0.2146	0.2259	0.3059	0.3797	0.2937	0.2128	0.1938
4Δd/d	0.0118	−0.0381	−0.1363	−0.1631	−0.1674	−0.1372	−0.0407

**Table 3:** FWHM index for raw material and thermal-modified products in the (002) reflection.

Samples	FWHM (°)
S_0_	0.137
S_1_ (500 °C)	0.138
S_1_ (600 °C)	0.168
S_1_ (700 °C)	0.173
S_1_ (800 °C)	0.174
S_1_ (900 °C)	0.141
S_1_ (1000 °C)	0.132
S_1_ (1100 °C)	-

**Table 4:** Relation between Bcosθ and sinθ of sericite with different holding time.

Holding Time (h)	Raw Material	1	2	3
kλ/D	0.2146	0.4287	0.2937	0.2277
4Δd/d	0.0118	−0.2848	−0.1674	−0.1026

**Table 5:** Design and results of the orthogonal experiment of acid treatment of sericite ^a^.

Trial No.	Factors				Results Dissolving-Out Amount of Al^3+^ (mg/g)
	Kinds of Acid *A*	Acid Concentration *B* (mol/L)	Reaction Temperature *C* (°C)	Reaction Time *D* (h)	
1	HNO_3_	1	60	1	4.2
2	HNO_3_	3	80	3	12.8
3	HNO_3_	5	95	5	31.0
4	H_2_SO_4_	1	80	5	9.9
5	H_2_SO_4_	3	95	1	14.0
6	H_2_SO_4_	5	60	3	8.2
7	HCl	1	95	3	11.1
8	HCl	3	60	5	8.5
9	HCl	5	80	1	9.9
K_1,j_	48.0	25.2	20.9	28.1	-
K_2,j_	32.1	35.3	32.6	32.1	-
K_3,j_	29.5	49.1	56.1	49.4	-
k_1,j_	16.0	8.4	7.0	9.4	-
k_2,j_	10.7	11.8	10.9	10.7	-
k_3,j_	9.8	16.4	18.7	16.5	-
R_j_	6.2	8.0	11.7	7.1	-

^a^: K_ij_ is defined as the sum of the evaluation indexes of all levels (i, i = 1, 2, 3) in each factor (j, j = A, B, C, D) and k_ij_ (mean value of K_ij_) is used to determine the optimal level and the optimal combination of factors. The optimal level for each factor could be obtained when k_ij_ is the largest; R_j_ is defined as the range between the maximum and minimum value of k_ij_ and is used for evaluating the importance of the factors.

**Table 6:** Design and results of the orthogonal experiment of sodium modification of sericite.

Trial No.	Factors			Results CEC (mmol/100 g)
	Concentration of Na^+^ *A* (mol/L)	Reaction Temperature *B* (°C)	Reaction Time *C* (h)	
1	1	60	1	15.76
2	1	80	3	27.24
3	1	95	5	29.70
4	3	60	3	25.36
5	3	80	5	35.24
6	3	95	1	40.76
7	Supersaturated	60	5	34.62
8	Supersaturated	80	1	42.34
9	Supersaturated	95	3	56.37
K_1,j_	72.70	75.24	98.86	-
K_2,j_	101.36	104.82	108.97	-
K_3,j_	133.33	126.83	99.56	-
k_1,j_	24.23	25.25	32.95	-
k_2,j_	33.79	34.94	36.32	-
k_3,j_	44.44	42.28	32.19	-
R_j_	20.21	17.03	4.13	-

**Table 7:** FWHM index for raw material and activate products in the (002) reflection.

Samples	FWHM (°)
S_0_	0.208
S_1_	0.226
S_2_	0.720
S_3_	0.452
